# Adenovirus-mediated delivery of bFGF small interfering RNA reduces STAT3 phosphorylation and induces the depolarization of mitochondria and apoptosis in glioma cells U251

**DOI:** 10.1186/1756-9966-30-80

**Published:** 2011-09-09

**Authors:** Jun Liu, Xinnv Xu, Xuequan Feng, Biao Zhang, Jinhuan Wang

**Affiliations:** 1 Graduate school, Tianjin Medical University (22# Qixiangtai road Hexi District), Tianjin(300070), China; 2 Department of Neurosurgery, Tianjin Huan Hu Hospital(122# Qixiangtai Road, Hexi District), Tianjin (300060), China; 3 Key Lab for Critical Care Medicine of the Ministry of Health, Tianjin First Center Hospital(24# Fukang road Nankai District), Tianjin (300192), China; 4 Department of Neurosurgery, Tianjin First Center Hospital(24# Fukang road Nankai District), Tianjin (300192), China; 5 Clinical Lab, Tianjin Huan Hu Hospital(122# Qixiangtai Road, Hexi District), Tianjin (300060), China

**Keywords:** bFGF, STAT3, IL-6, Glioblastoma multiforme

## Abstract

Glioblastoma multiforme (GBM) carries a dismal prognosis primarily due to its aggressive proliferation in the brain regulated by complex molecular mechanisms. One promising molecular target in GBM is over-expressed basic fibroblast growth factor (bFGF), which has been correlated with growth, progression, and vascularity of human malignant gliomas. Previously, we reported significant antitumor effects of an adenovirus-vector carrying bFGF small interfering RNA (Ad-bFGF-siRNA) in glioma *in vivo *and *in vitro*. However, its mechanisms are unknown. Signal transducer and activator of transcription 3 (STAT3) is constitutively active in GBM and correlates positively with the glioma grades. In addition, as a specific transcription factor, STAT3 serves as the convergent point of various signaling pathways activated by multiple growth factors and/or cytokines. Therefore, we hypothesized that the proliferation inhibition and apoptosis induction by Ad-bFGF-siRNA may result from the interruption of STAT3 phosphorylation. In the current study, we found that in glioma cells U251, Ad-bFGF-siRNA impedes the activation of ERK1/2 and JAK2, but not Src, decreases IL-6 secretion, reduces STAT3 phosphorylation, decreases the levels of downstream molecules CyclinD1 and Bcl-xl, and ultimately results in the collapse of mitochondrial membrane potentials as well as the induction of mitochondrial-related apoptosis. Our results offer a potential mechanism for using Ad-bFGF-siRNA as a gene therapy for glioma. To our knowledge, it is the first time that the bFGF knockdown using adenovirus-mediated delivery of bFGF siRNA and its potential underlying mechanisms are reported. Therefore, this finding may open new avenues for developing novel treatments against GBM.

## 1. Introduction

Glioblastoma multiforme (GBM) is the most common primary malignant brain tumor in adults. Despite technological advances in surgical resection followed by the application of combined radiotherapy and chemotherapy, GBM patients have a median overall survival of nearly one year [1,2]. A wide variety of genetic alterations that are frequently found in GBM are known to promote the malignant phenotype, including the abnormal activation of the PI3K-AKT and Ras-Raf-MEK-MAPK signaling pathways, the suppression of p53, retinoblastoma protein, and PTEN, as well as the amplification and/or alteration of epidermal growth factor receptor (EGFR) and vascular endothelial growth factor receptor (VEGFR) [3-5]. Basic fibroblast growth factor (bFGF), a heparin-binding polypeptide growth factor, exerts mitogenic and angiogenic effects on human astrocytic tumors in an autocrine way [6]. Overexpression of bFGF, but not of fibroblast growth factor receptor1, in the nucleus correlates with the poor prognosis of gliomas [7]. Thus, bFGF may be a promising target for novel therapeutic approaches in glioma. Previously, we reported that adenovirus-mediated delivery of bFGF small interfering RNA (Ad-bFGF-siRNA) showed antitumor effects and enhanced the sensitivity of glioblastoma cells to chemotherapy in glioma cell U251 [8,9]. However, the major mechanisms involved remain unknown.

Recently, the signal transducer and activator of transcription3 (STAT3) signaling pathway, which is constitutively activated in a variety of human neoplasms [10], such as leukemia, head and neck cancer, melanoma, breast cancer, prostate cancer, and glioma, has become a focal point of cancer research. In GBM, abnormally activated STAT3 activates a number of downstream genes to regulate multiple behaviors of tumor cells, such as survival, growth, angiogenesis, invasion, and evasion of immune surveillance. This aberrant STAT3 activation correlates with the tumor grades and clinical outcomes [11]. STAT3 can be activated by IL-6-family cytokines in the classic IL-6/JAK pathway [12,13] and by the growth factors EGF, FGF, and platelet-derived growth factor (PDGF) in target cells expressing receptor tyrosine kinases [14]. The oncoprotein Src can also directly activate STAT3 [15]. Given the fact that bFGF can activate the STAT3 pathway in many cell types, we investigated in this study whether the antitumor effects of Ad-bFGF-siRNA correlate with the reduced activation of the STAT3 signaling pathway to further our current understanding of the underlying mechanisms of Ad-bFGF-siRNA-induced growth suppression and apoptosis of glioma cells.

## 2. Materials and methods

### 2.1 Cell Culture and Adenovirus Infection

The human glioblastoma cell line U251 was cultured in Dulbcco's modified Eagle medium (DMEM) supplemented with 10% heat inactivated fetal bovine serum (FBS), 100 U/ml of penicillin, and 100 μg/ml of streptomycin in a humidified atmosphere containing 5% CO_2 _at 37°C. All media and serum were purchased from Gibcol. Normal human astrocytes (NHA) were obtained and maintained in specific growth medium AGM bullet kit from Clonetics-BioWhittaker (Walkersville, MD, USA).

U251 cells (2 × 10^5^) in serum-free DMEM were infected with Ad-bFGF-siRNA at 100 MOI or an adenovirus vector expressing green fluorescent protein (Ad-GFP) or null (Ad-null) as mock controls at 100 MOI. Cells treated with DMSO were used as the controls. 8 h later, the virus-containing medium was removed and replaced with fresh DMEM containing 10% FBS. Cells were further incubated for 24, 48, or 72 h, respectively. Cells were then lysed and total protein was extracted.

### 2.2 Western Blot

Western blot analysis was performed as previously described [8,9]. Briefly, the treated and untreated U251 cells were lysed in M-PER Reagent (Thermo Co, Ltd) containing the halt protease and phosphatase inhibitor cocktail. Protein (30 μg/lane), quantified with the BCA protein assay kit (Pierce, Fisher Scientific), was separated by 8-12% SDS-PAGE and transferred to PVDF membranes. The membranes were blocked with 5% non-fat dry milk in TBST (for non-phosphorylated proteins) or 5% BSA in TBST (for phosphorylated proteins) for 1 h and then incubated with primary antibodies overnight at 4°C. After washing, the membranes were incubated with secondary antibodies conjugated to horseradish peroxidase (1:5000) for 1 h at room temperature and developed by an ECL kit (Thermo Co., Ltd.)

### 2.3 Antibodies and regents

The primary antibodies were obtained from Santa Cruz (Beijing China) (bFGF, pJAK2 (Tyr1007/1008), STAT3, pSTAT3 (Ser727), CyclinD1, Caspase3, Cytochrome C, Bcl-xl, Bax, and Beta-actin). Other antibodies were form Genemapping (Tianjin China) (JAK2, pSTAT3 (Tyr705), anti-Src, anti-pSrc (Tyr419), anti-ERK1/2, anti-pERK1/2 (Thr202/Tyr204)). Human recombinant IL-6 was purchased from Sigma (Beijing China).

### 2.4 ELISA Analysis of IL-6 Release

The U251 cells were infected as above and collected from 0-24, 24-48, or 48-72 h periods IL-6 secretion was determined using a human IL-6 ELISA kit (4A Biotech, Beijing, China). The results were read using a microplate reader at 450 nm. A standard curve prepared from recombinant IL-6 was used to calculate the IL-6 production of the samples.

### 2.5 Measurement of mitochondrial transmembrane potential (ΔΨm)

Mitochondrial transmembrane potential (ΔΨm) was measured with the mitochondrial membrane potential assay kit with JC-1 (Beyotime, Shanghai, China). Cells were infected with Ad-bFGF-siRNA at 100 MOI for 8 h in 6-well plates, incubated in fresh DMEM for 72 h, and collected and resuspended in fresh medium. Cells were then incubated at 37°C for 20 min with 0.5 mL of JC-1 working solution. After that, the staining solution was removed by centrifugation at 600 g for 3-4 min and cells were washed twice with JC-1 staining 1 × buffer. Finally, cells were resuspended in 0.6 mL of buffer. At least 10,000 cells were analyzed per sample on the FACScaliber machine (BD Biosciences, San Jose, CA, USA). Additionally, ΔΨm was also observed by fluorescence microscopy. Briefly, untreated and treated cells were cultured in 6-well plates, stained with 1.0 mL of JC-1 working solution at 37°C for 20 min, washed twice with JC-1 staining 1 × buffer, and then observed using a fluorescence microscope at 200× (Olympus, Japan).

### 2.6 Statistical analysis

Results were analyzed using SPSS software 13.0 and compared using one-way analysis of variance (ANOVA). Data were presented as mean ± standard deviation (SD) of three independent experiments. *P *< 0.05 was considered statistically significant

## 3. Results

### 3.1 Ad-bFGF-siRNA reduces STAT3 phosphorylation at Ser727 and Tyr705 in a time-dependent manner in U251 cells

**Figure 1:**
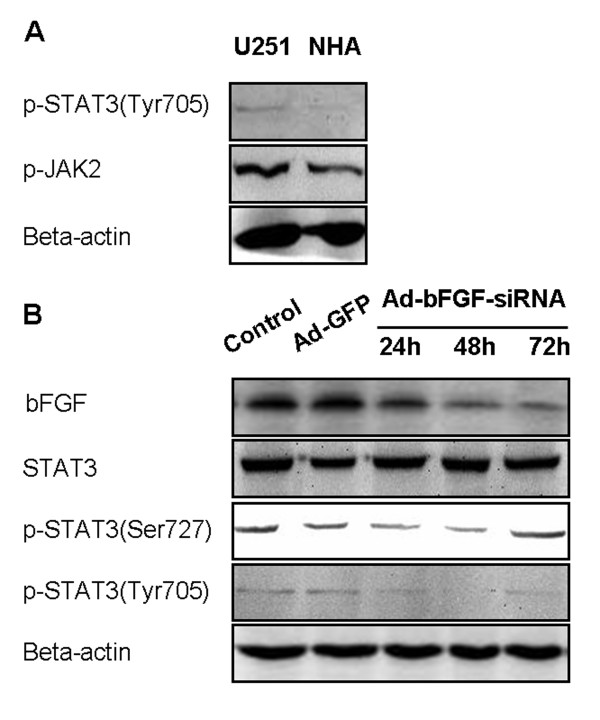
**Ad-bFGF-siRNA reduces STAT3 phosphorylation in U251 cells**. (A) Western blot analysis revealed that the levels of pSTAT3 (Tyr705) and pJAK2 are higher in U251 cells than in normal human astrocytes (NHA). (B) Ad-bFGF-siRNA (MOI = 100) reduces STAT3 phosphorylation (both Tyr705 and Ser727) in a time-dependent manner in U251 cells. Total STAT3 expression remains stable.

First, to investigate whether STAT3 and upstream kinases JAK1/2 are activated in U251 cells, we performed western blot and showed a higher expression of pSTAT3 Tyr705 and pJAK2 in the glioblastoma cell line U251 than in NHA (Figure 1A). The level of pJAK1 was not significantly elevated in U251 cells (data not shown).

Next, we knocked down bFGF using Ad-bFGF-siRNA, and the decrease in bFGF protein levels was confirmed by western blot (Figure 1B). Then, we examined whether Ad-bFGF-siRNA treatment affects STAT3 phosphorylation. STAT3 is fully activated when both of its two conserved amino acid residues Tyr705 and Ser727 are phosphorylated [16]. For this propose, we extracted total proteins from DMSO, Ad-GFP, and Ad-bFGF-siRNA treatment groups at 24, 48, and 72 h time points and examined the levels of total and phosphorylated STAT3 by western blot. The total STAT3 expression remained similar among three groups across different time points (Figure 1B). Interestingly, the expression of pSTAT3 Ser727 moderately decreased at 24 and 48 h and then restored to the control level at 72 h. Furthermore, compared with the levels under the control and Ad-GFP treatment, the level of pSTAT3 Tyr705 under Ad-bFGF-siRNA treatment was markedly decreased at all three time points, even to an undetectable level at 48 h point. Thus, these findings suggested that Ad-bFGF-siRNA interferes with the activation of STAT3 in a time-dependent manner and this decrease in pSTAT3 could not be explained by a constitutional decrease in total STAT3.

### 3.2 Ad-bFGF-siRNA reduces the activation of upstream kinases of the STAT3 signaling pathway and decreases the levels of downstream molecules

STAT3 is regulated by upstream kinases, including extracellular signal-regulated kinases (ERKs), JAKs, and non receptor tyrosine kinases, including Ret, Src, and the Bcl-Abl fusion protein [17]. Therefore, to better understand how the upstream cascade of STAT3 is affected by Ad-bFGF-siRNA in U251 cells, we examined the phosphorylation of ERK1/2, JAK2, and Src under Ad-bFGF-siRNA treatment.

**Figure 2:**
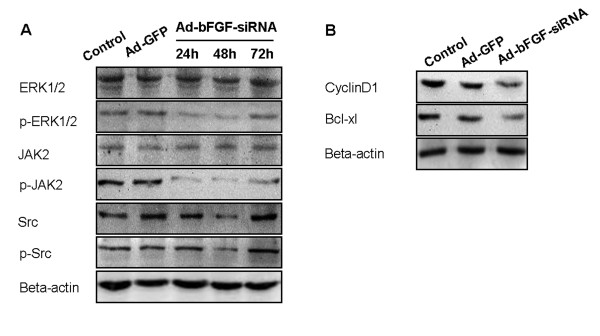
**Ad-bFGF-siRNA reduces the activation of upstream molecules and the expression of downstream molecules of STAT3 in U251 cells**. (A) Ad-bFGF-siRNA (MOI = 100) reduces the phosphorylation/activation of ERK1/2 and JAK2 in a time-dependent manner in U251 cells. Total ERK1/2 and JAK2 expression remains stable. Total and phosphorylated Src decreases at 48 h in a similar manner. (B) Ad-bFGF-siRNA (MOI = 100) reduces the expression of CyclinD1 and Bcl-xl at 72 h time point.

Interestingly, despite similar protein levels of total ERK1/2, when infected with Ad-bFGF-siRNA, the level of pERK1/2 decreased at 24 and 48 h compared with the levels in the Ad-GFP and control groups and increased to the control level at 72 h (Figure 2A). Similarly, while no change in total JAK2 was observed, the level of pJAK2 decreased at 24, 48, and 72 h time points (Figure 2A). In contrast, after bFGF knockdown, the total and phosphorylated Src decreased at 48 h in a similar manner, indicating that the phosphorylation/activation of Src is probably not affected by bFGF knockdown (Figure 2A).

To further explore the inhibition of STAT3 phosphorylation by Ad-bFGF-siRNA, we examined the levels of two downstream targets of STAT3: CyclinD1, which regulates cell cycle, and Bcl-xl, which is an important apoptosis-suppressor and is usually down-regulated in apoptotic cells. As shown in Figure 2B, at the 72 h time point, the levels of both CyclinD1 and Bcl-xl in the Ad-bFGF-siRNA group were significantly decreased compared with the levels in the Ad-GFP and control groups.

### 3.3 Correlation between pSTAT3 down-regulation and IL-6 secretion induced by Ad-bFGF-siRNA

**Figure 3:**
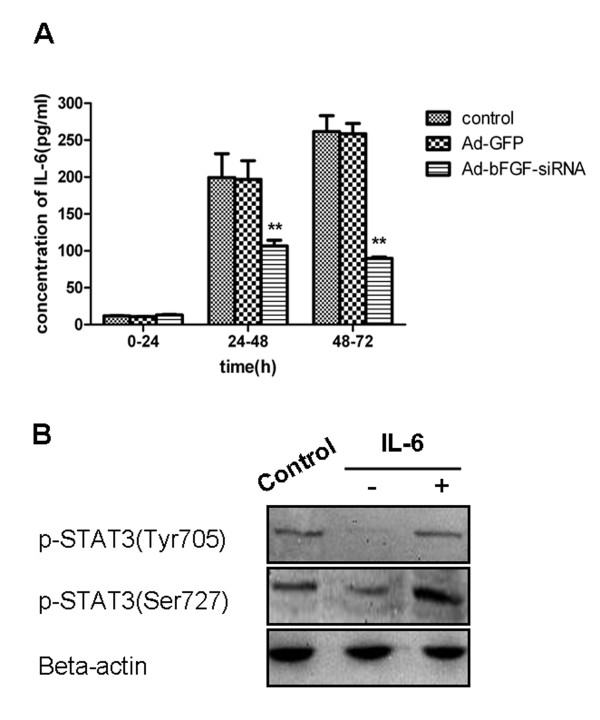
**Ad-bFGF-siRNA reduces IL-6 secretion in U251 cells**. (A) ELISA analysis showed that IL-6 secretion in the Ad-bFGF-siRNA group (MOI = 100) was lower than that in the control and Ad-GFP groups during both 24-48 h and 48-72 h periods. **: p < 0.0001. Data are presented as mean ± SD, n = 3. (B) U251 cells infected with Ad-bFGF-siRNA for 48 h were treated with serum-free DMEM in the presence or absence of recombinant IL-6 (100 ng/ml) for 24 h. Cells treated with DMSO for 72 h served as controls. The phosphorylation of STAT3 at both Tyr705 and Ser727 is elevated after stimulated with IL-6 for 24 h.

GBM cells secrete IL-6 both in an autocrine and localcrine way, and this IL-6 secretion is responsible for the persistent activation of STAT3 in GBM [18]. To examine whether Ad-bFGF-siRNA inhibits STAT3 phosphorylation by reducing IL-6 secretion, we tested the IL-6 level in the supernatant of U251 cells. The level of IL-6 was very low during the first 24 h and no significant difference was observed between the three groups (concentration in pg/mL: control: 11.93 ± 0.34; Ad-GFP: 10.92 ± 0.14; and Ad-bFGF-siRNA: 13.15 ± 0.74) (Figure 3A). During 24-72 h, the IL-6 level in the control and Ad-GFP groups increased markedly (24-48 h: control: 199.46 ± 32.11 and Ad-GFP: 196.99 ± 25.24; 48-72 h: control: 261.74 ± 21.47 and Ad-GFP: 258.50 ± 14.21) (Figure 3A). In contrast, the IL-6 level in the Ad-bFGF-siRNA group, although increased from that of the first 24 h, was significantly lower than that of the control and Ad-GFP groups (p < 0.0001; 24-48 h: 106.66 ± 7.70; 48-72 h: 89.87 ± 1.82) (Figure 3A). In conclusion, Ad-bFGF-siRNA inhibits IL-6 cytokine expression in a time-dependent manner.

To explore whether exogenous IL-6 can rescue Ad-bFGF-siRNA-inhibited STAT3 activation, U251 cells infected for 48 h were treated with serum-free DMEM in the presence or absence of recombinant IL-6 (100 ng/ml) for 24 h. Cells treated with DMSO for 72 h were used as a negative control. As shown in Figure 3B, the phosphorylation of STAT3 at both Tyr705 and Ser727 was elevated after stimulated with IL-6 for 24 h.

### 3.4 Ad-bFGF-siRNA induces depolarization of mitochondria and apoptosis in U251 cells

**Figure 4:**
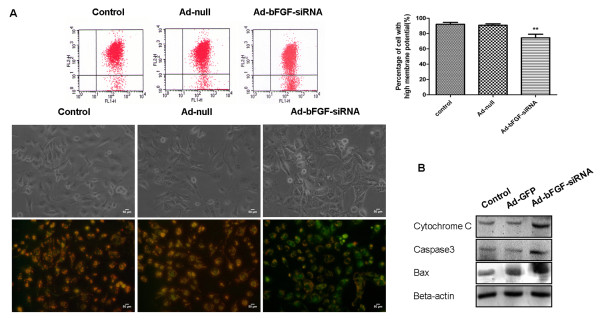
**Ad-bFGF-siRNA reduces the mitochondrial transmembrane potential (ΔΨm) and induces apoptosis in U251 cells**. (A) Cytofluorimetric analysis using JC-1 staining demonstrated that Ad-bFGF-siRNA treatment (MOI = 100) induces depolarization of mitochondria. Percentages of cells with high ΔΨm (%) are shown in each column. Data are represented as mean ± SD of three replicates (**: P < 0.0005). Changes in ΔΨm were also detected by fluorescence microscopy. Magnification: 200×. Scale bar: 50 μm. Normal cells that have high ΔΨm show punctuate yellow fluorescence. Apoptotic cells show diffuse green fluorescence because of the decrease in mitochondrial membrane potential. (B) Western blot analysis revealed that Ad-bFGF-siRNA (MOI = 100 for 72 h) increases the expressions of Cytochrome C, Caspase3, and Bax.

Given the central role of mitochondria in orchestrating the apoptotic processes, we assessed the mitochondrial transmembrane potential (ΔΨm) after bFGF knockdown by Ad-bFGF-siRNA using JC-1 staining. JC-1 forms high orange-red fluorescent J-aggregates (FL-2 channel) at hyperpolarized membrane potentials and weak green fluorescent monomers (FL-1 channel) at depolarized membrane potentials. The results showed that the control and Ad-Null cells exhibited high orange-red fluorescence and weak green fluorescence (Figure 4A), indicating hyperpolarized mitochondria. In contrast, after treated with Ad-bFGF-siRNA (MOI = 100) for 72 h, an increased subpopulation of cells displayed decreased orange-red fluorescence, suggesting the collapse of mitochondrial membrane potentials. The ratio of cells with high membrane potentials in the Ad-bFGF-siRNA group (90.87 ± 1.84%) decreased significantly from that in the control and Ad-Null groups (92.12 ± 2.50% and 74.42 ± 4.66%, respectively; p < 0.0005)

Furthermore, to reveal whether apoptosis is triggered by Ad-bFGF-siRNA, we examined the levels of three important players in apoptosis: Cytochrome C, Caspase3, and Bax. As shown in Figure 4B, the level of Cytochrome C, Caspase3, and Bax was markedly higher in the Ad-bFGF-siRNA group than in the control and Ad-GFP groups, confirming the activation of apoptosis under Ad-bFGF-siRNA treatment.

## 4. Discussion

Recent studies have demonstrated that over-activation of STAT3 is observed in several human malignant tumors and cell lines, including glioblastoma [19,20]. Abnormal and constitutive activation of STAT3 may be responsible for glioma progression through regulating the expression of target genes, such as CyclinD1, Bcl-xl, IL-10, and VEGF, whereas functional inactivation of STAT3 by dominant-negative STAT3 mutants inhibits proliferation and induce apoptosis of glioma [21]. Since STAT3 is activated by cytokine receptor-associated tyrosine kinases or growth factor receptor intrinsic tyrosine kinases, besides antagonizing the function of relevant kinases or receptors, targeting the over-expressed ligands that inappropriately stimulate the activation of STAT3 is also a promising strategy for glioma [22].

In this study, we provided evidence that Ad-bFGF-siRNA can inhibit the phosphorylation of STAT3 by down regulating the activation of ERK1/2 and JAK2, but not Src signaling transduction (Figure 1 and 2). This inhibition of STAT3 phosphorylation/activation subsequently down-regulates downstream substrates of STAT3 and induces mitochondria-related apoptosis in U251 cells (Figure 2 and 4). Importantly, the aberrant expression of IL-6 in GBM cells is also interrupted by Ad-bFGF-siRNA (Figure 3), which could be a potential mechanism for Ad-bFGF-siRNA to serve as a targeted therapy for glioma *in vitro *and *in vivo*.

bFGF exerts functions via its specific binding to the high affinity transmembrane tyrosine kinase receptors [23] and the low affinity FGF receptors (FGFR1-4) [24]. The binding of bFGF by FGFRs causes dimerization and autophosphorylation of receptors and subsequently activates serine-threonine phosphorylation kinases such as Raf, which triggers the classic Ras-Raf-MEK-MAPK (ERK) signaling pathway [25]. As a central component of the MAPK cascade, over-activated ERK1/2 contributes to malignant transformation [26]. After ERK1/2 is phosphorylated and dimerized, it translocates into the nucleus and phosphorylates an array of downstream targets, including STAT3 [27]. Previously, it has been reported that FGF-1 stimulation leads to the activation of ERK1/2, which in turn phosphorylates STAT3 at Ser727 in prostate cancer cells [28]. In addition, bFGF has been shown earlier to activate ERK and phosphorylate STAT3 at Tyr705 in myoblasts [29]. However, it remains unknown what happens in glioma. In our study, we applied bFGF knockdown and demonstrated that STAT3 phosphorylation at both Tyr705 and Ser727 is reduced by Ad-bFGF-siRNA treatment in a time-dependent way (Figure 1B). In agreement with the down-regulation of pSTAT3 Ser727, the activation of ERK1/2 was also decreased in a similar manner (Figure 2A), indicating that bFGF knockdown probably inhibits the ERK1/2 cascade, which in turn down-regulates STAT3 phosphorylation at Ser727.

IL-6 is a critical tumor promoter regulated by activated transcription factor NF-κB [30] and IL-6 gene amplification occurs in 40-50% of GBM patients [31]. Due to its ability to activate STAT3, the elevated IL-6 and its family members have been strongly implicated in GBM [32]. Interestingly, Ad-bFGF-siRNA down-regulates IL-6 expression possibly through inhibiting NF-κB activation. This IL-6 down-regulation may be responsible for the reduced activation of STAT3 at Tyr705 [33]. Indeed, IL-6 supplementation restores the level of pSTAT3 Tyr705 after 24 h incubation (Figure 3B). Surprisingly, exogenous IL-6 also elevates the level of pSTAT3 Ser727 (Figure 3B) and future studies are required to examine the underlying mechanisms.

To determine the potential mechanism of STAT3 inactivation, the activation of the JAK2-STAT3 pathway was examined. Upon stimulation with growth factors, such as EGF and PDGF, or IL-6 family cytokines, JAK2 proteins bind receptors and trans- or auto-phosphorylate themselves as well as the cytoplasmic tail of the receptors. Subsequently, STAT3 is tyrosine phosphorylated and homodimerizes or heterodimerizes with STAT1 [34]. In addition, c-Src, as a key non-receptor tyrosine kinase, can directly phosphorylate the tyrosine residues of STAT3 through the SH-2 domain independent of JAK [35]. Src exhibits a high expression level in the nervous system and plays an important role in the deregulated proliferation and uninhibited growth of brain tumors [36]. STAT3 activation by bFGF-FGFR binding has been implicated in the regulation of JAK2 and Src kinase activities in human umbilical vein endothelial cells [37]. However, little has been reported on the effects of inhibiting bFGF expression on the JAK2-STAT3 pathway in glioma. Our results showed the down-regulation of bFGF inhibits the phosphorylation of JAK2 at 24, 48, and 72 h time points (Figure 2A). In contrast, the phosphorylation/activation of Src is not affected by bFGF knockdown. In conclusion, Ad-bFGF-siRNA interferes with the JAK2-STAT3 signaling pathway in a time-dependent way, but exerts no effect on Src phosphorylation.

The decrease in STAT3 activation by Ad-bFGF-siRNA can induce multiple effects in glioma cells U251. Our results showed the STAT3 downstream factor CyclinD1 was diminished (Figure 2B). Since we observed no cell cycle arrest during the Ad-bFGF-siRNA treatment [9], the proliferation inhibition by Ad-bFGF-siRNA may be due to proapoptotic effects rather than cell cycle arrest. Concomitantly, the elevated Caspase3, Bax, and Cytochrome C levels (Figure 4B) and the reduced Bcl-xl levels (Figure 2B) may underlie the antitumor effects of Ad-bFGF-siRNA. Furthermore, as a sign of early apoptosis, ΔΨm is also decreased after Ad-bFGF-siRNA treatment (Figure 4A). Bcl-2 and Bcl-xl counteract the proapoptotic effects of Bax and Bcl-2 antagonist killer and inhibit the mitochondria-mediated cell death pathway [38]. Once the expression of Bcl-2 and/or Bcl-xl decreases, elevated Bax translocates to the mitochondria membrane, induces the opening of the mitochondrial permeability transition pore (PTP) to release Cytochrome C and causes mitochondria-dependent apoptosis. Here, we showed that Ad-bFGF-siRNA antagonizes the STAT3 pathway activation and depolarizes membrane potentials to induce depolarization of mitochondria and apoptosis in U251 cells.

In conclusion, as one of the new avenues in gene therapy, siRNA has emerged as a great potential for the treatment of glioma. The adenovirus-mediated delivery of bFGF siRNA presents one such promising approach and the current data provide a mechanistic explanation for this novel strategy. Future studies are needed to test its efficacy in other glioma cell lines such as U87 and U138 cells to further corroborate the current findings.

## Competing interests

The authors declare that they have no competing interests.

## Authors' contributions

JL carried out experiments and drafted the manuscript. XX participated in study design and helped to draft the manuscript. XF and BZ participated in study design, performed experiments and JW participated in study design and revised manuscript. All authors approved the final manuscript.
